# Human rights violations among men who have sex with men and transgender people in South Africa

**DOI:** 10.4102/sajhivmed.v24i1.1417

**Published:** 2023-01-23

**Authors:** Raymond Chimatira, Dumo Jebese-Mfenqe, Joram Chikwanda, Edward Sibanda, Qhawekazi Thengwa, Bulumko Futshane, Sisanda Gaga

**Affiliations:** 1 Beyond Zero, East London, South Africa

**Keywords:** HIV, human rights violations, key populations, men who have sex with men, transgender people, South Africa

## Abstract

**Background:**

Men who have sex with men (MSM) and transgender (TG) people face human rights violations (HRVs) which impact their access to critical interventions for HIV prevention, treatment, and related services.

**Objectives:**

This study describes how Beyond Zero, a not-for-profit organisation in South Africa, built an HRV reporting system and discusses data on the HRVs experienced by MSM and TG people who accessed HIV prevention services between 01 January 2021 and 31 December 2021.

**Method:**

This was a cross-sectional study using secondary analysis of programmatic data routinely collected as part of HIV prevention programmes for MSM and TG in 10 rural districts of South Africa.

**Results:**

A total of 249 individuals reported having experienced HRVs. Of these, 113 (54.6%) were physical violations, 145 (58.2%) were psychosocial harassment, 15 (18.3%) were experienced within the workplace, and 59 (23.7%) were experienced at a healthcare or social services institution. Overall, 77% of the physical violations and 70.4% of the psychosocial violations occurred in the home and local community settings; 76.1% of the perpetrators of physical violence and 79.3% of the perpetrators of psychosocial harassment were known. Most incidents of physical violence (80.5%) and psychosocial harassment (92.4%) were not reported due to fear of homophobic or transphobic violence.

**Conclusion:**

Our findings demonstrate the feasibility of documenting HRVs among MSM and TG people within HIV prevention programmes. Men who have sex with men and TG people should be systematically screened for HRVs and linked to legal or other services.

**What this study adds:**

Our findings present data on the nature of HRVs in 10 districts outside of the large urban centres where research documenting the lived experiences of MSM, TG people and other key populations is traditionally conducted in South Africa. This data contribute to addressing the gap in the literature on the needs of MSM and TG people in South Africa caused by the delayed inclusion of rural MSM and TG people in research.

## Introduction

South Africa has one of the highest burdens of HIV globally, with an estimated 7 800 000 people living with HIV in 2020. While South Africa has a generalised HIV epidemic, the prevalence rates are highest among key populations.^1,2^ Uptake of HIV-related treatment and prevention services and retention in care also varies, with lower rates reported among sex workers, men who have sex with men (MSM) and other vulnerable populations. Human rights violations (HRVs) affect this situation by driving the HIV epidemic, contributing to significant vulnerabilities to new infections and presenting barriers to access to HIV prevention and treatment services.^3,4,5^ Consequently, this article focuses on documenting HRVs within the context of comprehensive HIV prevention programmes for MSM and transgender (TG) people in 10 districts in South Africa.

Stigma, discrimination, gender inequality and socio-cultural norms that drive physical and sexual violence and psychosocial harassment against key populations create barriers to accessing healthcare and social services, and negatively affect retention in care for the very populations most in need.^3,4,6,7,8,9^ These violations also negatively affect employment opportunities and relationships in their communities. To address these disparities, public health experts and funding agencies have redefined the right to health to include creating an environment that affirms the dignity of key and vulnerable populations. In addition, funding agencies have increased investment in programmes to remove human rights-related barriers to HIV-related prevention and treatment services.^3,7,9,10,11^

The National Strategic Plan (NSP) for HIV, tuberculosis (TB) and sexually transmitted infections (STIs): 2017–2022 recognises that:

there are still important gaps to close with respect to the full implementation of the human rights agenda, particularly the rights of people living with HIV and TB and key and vulnerable populations.^8^ (p. 32)

Goal 5 of the NSP aims to ground the response to HIV, TB and STIs in human rights principles and approaches, to reduce stigma and discrimination, ensure equal treatment for all and increase access to justice in the context of HIV, TB and STIs for all vulnerable and key populations.^2,8^

The NSP 2019–2022 sets out key programmes to reduce human rights-related barriers to HIV and TB services and gender inequality in South Africa. Several interventions are implemented by governmental, non-governmental and private sector stakeholders to ensure the protection and promotion of HIV-related human rights in the country. It is against this background that Beyond Zero (BZ), a not-for-profit organisation in South Africa, developed a system for recording and responding to HRVs against MSM and TG people to strengthen its human rights programming. This study aimed to describe the nature of HRVs experienced by MSM and TG people within the context of comprehensive HIV prevention programmes for MSM and TG people in 10 districts in South Africa.

## Methods

### Study design

We conducted a cross-sectional study using secondary analysis of routinely collected programmatic data under the MSM and TG comprehensive HIV prevention programmes implemented by BZ through sub-recipients (SRs). We used both quantitative and qualitative methods. Quantitative data were collected by determining the number of verified violations reported to BZ by programme staff from the implementing SRs from 01 January 2021 to 31 December 2021. Qualitative data were collected by considering the cases of MSM and TG people reported to BZ by SRs in respect of the nature of the violations and factors surrounding the violations, such as the identity of perpetrators, the location where the violations occurred, and the steps taken by SRs to address reported violations.

### Study setting

**FIGURE 1:**
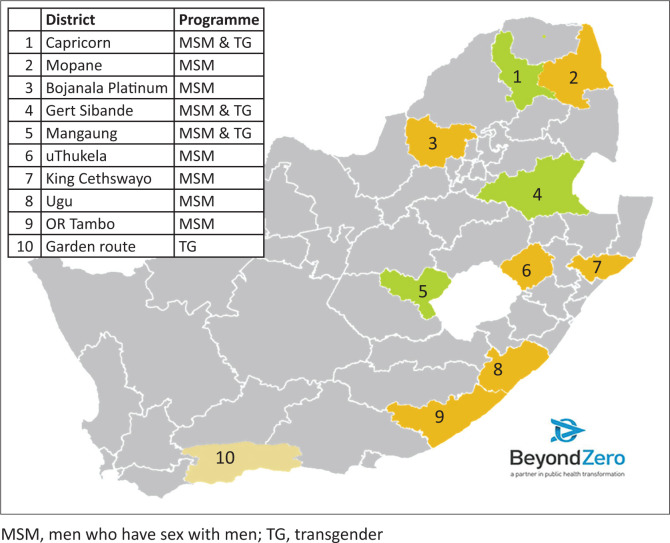
Districts implementing the Beyond Zero men who have sex with men and transgender programmes, Global Fund Grant 2019–2022.

Beyond Zero implemented the MSM comprehensive HIV prevention programme in nine districts through five SRs and the TG comprehensive HIV prevention programme in four districts through four SRs during the grant period 01 April 2019 to 31 March 2022. Figure 1 highlights the geographic spread of the MSM and TG programmes across South Africa.

The comprehensive HIV prevention programmes reached 76 084 MSM and 5337 TG people with a defined package of HIV prevention services (REACH Indicator) from 01 January 2021 to 31 December 2021. The REACH indicator is defined as the number of MSM who have received the defined minimum package of services, which includes all of the following components:

- Peer education inclusive of comprehensive sexual and reproductive health information.
- Enrolment risk assessment per individual, including HIV risk and STI, TB and gender-based violence screening, risk reduction counselling.
- Offered reasonable access to condoms and lubricants, either directly provided by peer educators or made available through dispensing machines in hotspots or clinics.
- Offered an HIV test if deemed relevant during the risk assessment. Assisted HIV self-screening may be offered, in addition to traditional HIV rapid testing.

### Intervention description – Screening, documenting, and reporting human rights violations

In the absence of a national system to systematically document and report HRVs among key populations, BZ used results from a desktop review of NSP, national policies, and the national stigma index to develop the HRV Documentation and Reporting System between October 2020 and December 2020. During this time, the Programme Management Team and Strategic Information Unit created the HRV documentation forms, developed the system’s electronic data capture forms and determined how the system would integrate with existing BZ reporting and data management systems. In addition, the team identified how to address user feedback and trained SR staff on how to screen for and document HRVs among MSM and TG people reached by the comprehensive prevention programmes.

**FIGURE 2:**
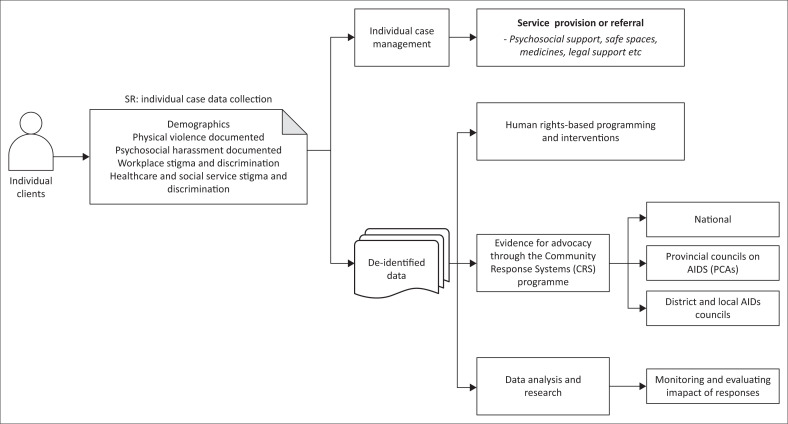
Conceptual framework for Beyond Zero’s human rights monitoring and reporting system.

The HRV monitoring and reporting system is based on a conceptual framework that outlines the following: (1) the necessary activities at the SR level, (2) individual case management strategies, (3) individual-level data collected at the SR level, (4) the use of de-identified data to improve programme implementation, and (5) mechanisms for reporting de-identified data to inform policy and action. Figure 2 outlines a high-level summary of the conceptual framework.

While the system could potentially allow individuals to report the HRVs online, the initial phase focused on the SR staff screening for and documenting HRVs experienced by MSM and TG people using an opt-out approach. The approach of in-person screening conducted by trained SR staff allowed the respective service providers to provide or refer individuals who experienced HRVs for psychosocial, legal and other related support services.

To improve data security and maintain client confidentiality while documenting HRVs, BZ and SRs avoid collecting identifying information in paper or electronic forms. At a minimum, the SRs collect contact information on a separate de-linked form (e.g. client records) with the client’s unique identification number to track service provision or referral for off-site services. In addition, the SRs allow clients to provide information in a private space and only report de-identified data to BZ. These data are stored on a secure server with encryption (SurveyCTO^®^), with user access control. Beyond Zero has no access to any personally identifiable information that could potentially be used to identify a particular person. In addition, the units of analysis are the districts, which are large enough that it will not be possible to infer information about individuals based on aggregate reports.

Once a case is documented, each SR provides direct psychosocial support or other appropriate services or linkage to care and support at other organisations or institutions that are better equipped to handle them. For example, SRs refer assault cases to the South African Police Services (SAPS) and work with legal service organisations to ensure their legal cases are reported to appropriate authorities.

Beyond Zero launched the system in December 2020 and conducted two primary interventions between the launch and January 2021. First, BZ trained SR staff to sensitively manage cases of discrimination and HRVs against MSM and TG people and use the reporting system. Second, BZ strengthened the internal relationships between the MSM and TG programmes with the Community Response and Systems Strengthening programme. The linkage with the Community Response and Systems Strengthening module allows for efficient dissemination of the reports and findings among the provincial and district stakeholders, for example provincial councils on AIDS, district AIDS councils, local AIDS councils, and civil society sector representatives.

These data are also reported to the Aids Foundation South Africa (AFSA), which was coordinating the Global Fund human rights programme for all the implementing principal recipients in South Africa (2020–2022). These data are merged with other HRV data collected by all principal recipients and disseminated to various key national stakeholders during quarterly operational programmatic excellence and coordination meetings. The key stakeholders include the South African National AIDS Council (SANAC), the Global Fund County Team, other development partners such as the United States (US) President’s Emergency Plan for AIDS Relief (PEPFAR), the US Centers for Disease Control and Prevention (CDC), and US Agency for International Development (USAID) and other non-governmental implementing partners.

### Data collection instrument and case definitions

For purposes of monitoring and documenting HRVs, BZ developed a detailed screening form through iterative rounds of inputs from programme technical leads and feedback from SR staff, some of whom identify as MSM or TG (Online Appendix 1). Recall of the HRVs experienced and characteristics of the perpetrators were elicited during risk assessment. Table 1 summarises the key questions and case definitions developed following a literature review.

**TABLE 1:** Key variables of the data collection instrument.

Section	Nature of violation	Key questions and case definition
A	Physical violence	In the last month, have you been subjected to any physical violence, as defined below, because you were known or suspected to be gay, bisexual, having sex with other men, or transgender? This includes being repeatedly hit or kicked, pushed, punched, pulled, being assaulted with a weapon, sexually harassed (e.g. touched against your will), sexual assault (e.g. rape or rape attempt), or other (specify).
B	Psychological harassment	In the last month, have you been subjected to any psychological harassment, as defined below, because you were known or suspected to be gay, bisexual, a man having sex with other men or a transgender person? This includes insults, humiliation, ridicule, malicious gossip, threats, being ostracised, hate mail, blackmail, damage to or theft of property, or other (specify).
C	Workplace stigma and discrimination	For clients indicating that in the past 12 months, they had held a full-time or part-time job or searched for work at any time. In the past 12 months, have you experienced any of the following situations in the workplace or while searching for work because you were known to be or suspected of being gay or bisexual or transgender or having sex with other men? Refusal of employment; refusal of promotion; dismissal; higher expectations in comparison to other employees or candidates; or none of these. In the past 12 months, have you felt the need to conceal your sexual orientation or avoid discussing it at the workplace or while searching for work?
D	Stigma and discrimination at health or social service institutions	In the past 12 months, have you experienced a situation where a representative (e.g. at educational institutions, healthcare facilities, home affairs services or other social services), having learned about your sexual orientation or identity, treated you differently or less favourably than before or than heterosexual patients?

### Data collection and analysis

The data from SRs were captured electronically into an online SurveyCTO^®^ form. The authors used SurveyCTO^®^’s built-in *Data Explorer* to summarise the data submitted for individual fields, summarise the empirical relationships between fields, and drill down to browse individual submissions. The qualitative responses were analysed using thematic content analysis.

### Ethical considerations

This study involved secondary analysis of routinely collected HIV prevention programme data that were collected as part of routine programme service delivery. Beyond Zero obtained ethical clearance for this study from the Pharma-Access Health Research Ethics Committee (Ethical Clearance Reference No. 210223835).

## Results

### Sociodemographic characteristics of individuals experiencing human rights violations

Between 01 January 2021 and 31 December 2021, SRs reported that 249 individuals reported having experienced HRVs. Overall, participants were young, with a mean age of 26 years (median 25 years). With regard to sexual orientation, 115 (46.2%) identified as men who have sex with men. With regard to gender identity, 29 (11.7%) identified as TG women, 18 (7.2%) identified as TG men, 42 (16.9%) were gender non-conforming and 45 (18.1%) preferred not to say. The majority, 138 (55.4%), had at least a secondary school education (Grades 8 to 12), and 167 (67.1%) were unemployed. Mopani, King Cetshwayo and Capricorn districts accounted for 49.5% of the reported violations. The variation in the number of HRVs reported reflects differences in uptake and rollout of the intervention by the SRs.

### Human rights violations reported

Of the 249 individuals who reported experiencing HRVs during the period 01 January 2021 to 31 December 2021, 145 (58.2%) experienced psychosocial harassment, 113 (45.4%) experienced physical violations, 15 (18.3%) experienced HRVs while at the workplace or applying for work, and 59 (23.7%) experienced HRVs at a healthcare or social services institution (Table 2). The most common types of violations reported (Table 3) were verbal insults (47.4%), psychosocial humiliation (35.7%), physical assault (26.9%), psychosocial ridicule (16.9%), malicious gossip (16.1%) and sexual assault (14.5%).

**TABLE 2:** Selected characteristics of men who sex with men and transgender who experienced human rights violation.

Descriptive variables	Overall	
	*n*	%
**Sex assigned at birth**		
Male	207	83.1
Female	36	14.5
Prefer not to say	6	2.4
**Education**		
Primary (Grades 1–7)	14	5.6
Secondary (Grades 8–12)	138	55.4
Tertiary (technical and vocational, university or other post-matric)	93	37.3
Prefer not to say	4	1.6
**Have you been at work at any time during the year?**		
No	167	67.1
Yes	82	32.9
**In the last month, have you been subjected to any of the following because you were known or suspected to be gay, bisexual, or having sex with other men, or a transgender person?**		
**Physical violence**		
Yes	113	54.6
No	136	45.4
**Psychosocial harassment**		
Yes	145	58.2
No	104	41.8
**For those who have worked in the past 12 months (*n* = 82): have you experienced any stigma or discrimination at the workplace or while applying for work?**		
Yes	15	18.3
No	67	81.7
**In the past 12 months, have you experienced a situation where a representative at a social services institution, having learnt about your sexual orientation or gender identity, treated you differently or less favourably than before or than heterosexual ind ividuals?**		
Yes	59	23.7
No	155	62.3
No, because I have not sought any services, or I conceal my sexual orientation or gender identity	35	14.0
**Reporting district, province**		
Mopani, Limpopo	43	17.3
King Cetshwayo, KwaZulu-Natal	40	16.1
Capricorn, Limpopo	40	16.1
uThukela, KwaZulu-Natal	33	13.3
OR Tambo, Eastern Cape	33	13.3
Mangaung, Free State	20	8.8
Bojanala, North West	14	5.6
uGu, KwaZulu-Natal	11	4.4
Gert Sibande, Mpumalanga	8	3.2
Garden Route, Western Cape	7	2.8

Note: Age at last birthday: mean (years) = 26, standard deviation = 6.62%.

**TABLE 3:** Commonly reported violations.†

Type of violation	*n*	%
**A. Physical violations**	113	54.6
Physical assault (e.g. repeatedly hit or kicked; or assaulted with a weapon)	67	29.9
Sexual assault (e.g. rape or rape attempt)	36	14.5
Sexual harassment (e.g. touched against one’s will)	24	9.6
Other	12	4.8
**B. Psychosocial harassment**	145	58.2
Verbal insults	118	47.4
Psychosocial humiliation	89	35.7
Ridicule	42	16.9
Malicious gossip	40	16.1
Threats	32	12.9
Damage to or theft of property	11	12.9
Ostracised	9	3.6
Hate mail	9	3.6
Blackmail	8	3.2
Other	5	2.0
**C. Workplace violations**	15	18.3
Refusal of employment	5	2.0
Refusal of promotion	3	1.2
Dismissal	2	0.8
Higher expectations in comparison to other employees or candidates	2	0.8
**D. Stigma and discrimination while accessing healthcare or social services**	59	23.7
Stigmatised within healthcare facilities (e.g. insults; poor quality of services)	15	6.0
Stigmatised within educational institutions (school**s** or universities)	12	4.8
Stigmatised within places of worship (e.g. refusal of entry; insults)	12	4.8
Stigmatised at the Department of Home Affairs	4	1.6

† , Some individuals reported experiencing multiple forms of human rights violations. These were all documented and form part of the descriptive analysis.

### Location where human rights violations experienced and the identity of the perpetrators

A review of the reported cases revealed that 51.3% of the physical violations occurred in the home, followed by 25.7% occurring on the streets within the local community. In comparison, the psychosocial violations occurred equally in the home (34.5%) and the streets within the local community (35.9%). Other incidents of physical attacks occurred in bars or nightclubs (15.9%), educational institutions (8.9%), workplaces (4.4%), parks (0.9%) and public transport (0.9%). Other incidents of psychosocial harassment occurred at educational institutions (10.3%), bars or nightclubs (5.5%), workplaces (4.8%), public transport (3.5%) and shopping malls or similar (2.1%).

About a quarter (*n* = 59) of the 249 individuals reported experiencing stigma and discrimination at health or social service institutions in the previous 12 months based on sexual orientation or sexual identity. While 155 (62.2%) did not experience violations when seeking healthcare or other social services, 35 (14.1%) had not utilised any healthcare or social service institutions or concealed their sexual orientation. Of 82 (32.9%) beneficiaries who were employed in the previous 12 months, 15 (18.3%) experienced stigma and discrimination in the workplace. In addition, 31 (37.86%) felt the need to conceal their sexual orientation from some of their work colleagues, while 8 (9.8%) concealed their sexual orientation all the time.

The data highlight that 77.0% of the physical violations and 70.4% of the psychosocial violations occurred in the home and on the streets within the local communities. As a result, 76.1% of the perpetrators of physical violence and 79.3% of the perpetrators of psychosocial harassment were known by the individuals experiencing HRVs (Table 4).

**TABLE 4:** Identity of the perpetrators of human rights violations.

Identity of the perpetrator	Physical HRV		Psychosocial	
	*N*	%	*N*	%
Friends or acquaintances	28	23.1	37	25.5
Family members (e.g. biological or step-parents, siblings, close relatives)	26	22.2	32	22.1
Intimate partners	16	13.7	6	4.1
Fellow students or teachers	9	7.7	14	9.7
Co-workers	3	2.6	5	3.4
Police officers	2	1.7	0	0.0
Other (e.g. neighbours, local community members)	7	6.0	21	14.5
Unknown	27	23.1	30	20.7

HRV, human rights violations.

### Barriers to reporting physical and psychosocial violations

All individuals who reported experiencing HRVs where asked if they had reported these violations to the SAPS or any relevant authority (e.g. institutional heads). For those who did not report to SAPS or other relevant authorities, the reasons for not reporting were elicited using predefined categories, with allowance for other reasons to be specified as free text.

While 113 individuals (54.6%) experienced physical violence, 91 (80.5%) incidents were not reported to the SAPS or any relevant authority (e.g. institutional heads). Around half, 48 (52.6%), did not report the incident due to fear of homophobic or transphobic violence, 14 (15.4%) felt the incident was not severe enough, and 8 (8.8%) felt that SAPS were not effective enough or no mechanism would recognise the discriminatory motive of an incident. A further 21 (23.1%) cited other reasons such as fear of being outed and having to come out in public (fear of embarrassing the family and intimate partners), preferring mediation within the family or community, and fear of losing benefits (employment or depended on the abuser for livelihood).

While 145 individuals (58.2%) experienced psychosocial violence, 134 (92.4%) incidents were not reported to the authorities. Of these, 50 (37.3%) did not report the incident due to fear of homophobic or transphobic violence, 45 (33.6%) felt the incident was not severe enough, and 10 (7.4%) felt that SAPS were not effective enough or no mechanism would recognise the discriminatory motive of an incident. Moreover, 29 (21.6%) cited other reasons such as fear of being outed and having to come out in public (fear of embarrassing the family and intimate partners), preferring mediation within the family or community, and fear of losing benefits (employment or depended on the abuser for livelihood).

## Discussion

We analysed routinely collected programmatic data to examine the nature of HRVs among MSM and TG people in 10 districts in South Africa. The individuals who experienced HRVs in our programmes were mainly young MSM and TG people with a mean age of 26 years and a fair level of education (92.8% had secondary education or higher). Our findings are similar to observations in several sub-Saharan African countries that young MSM and TG people are especially vulnerable to HRVs.^6,12,13,14,15,16^

Most perpetrators of physical violence and psychosocial harassment were known by the individuals experiencing HRVs, as these occurred in the home and on the streets within the local communities. Similar findings were reported among MSM in Tanzania, with verbal and moral abuse being the most prevalent from people in the street, neighbours and family members.^13^ In addition, there is evidence that sexual behaviour stigma at a community level is associated with individual-level risk behaviours among MSM and TG people.^6,17,18^ These high rates of HRVs by known perpetrators in the home and community, as well as the association between homophobic behaviour and individual-level risk behaviours, support the need for evidence-informed community-level interventions addressing stigma and discrimination in a culturally sensitive manner.

Of particular interest is that most incidents of physical violence (80.5%) and psychosocial harassment (92.4%) were not reported for mediation or action: 52.6% of the incidents of physical violence and 37.3% of the incidents of psychosocial harassment were not reported due to fear of homophobic or transphobic violence. These findings suggest that while South Africa has a progressive constitution protecting individual rights, the legal framework is insufficient to safeguard MSM, TG people and other key populations who continue to face stigma and discrimination while accessing healthcare and other social services. While not explored in our initial phase of the project, there is evidence that community-level homophobia and concealment of sexual orientation or sexual identity impact mental health, affecting access to, uptake of and retention in HIV prevention services.^6,15,18^

Our findings support the need for legal and social change interventions to change attitudes regarding sexual minorities and address stigma, discrimination and HRVs affecting MSM, TG people and other key populations. This requires the rapid scale-up and monitoring of the implementation plan outlined in the NSP to reduce human rights-related barriers to HIV and TB services in South Africa 2019–2022. The implementation plan outlines seven critical programme areas to address HIV, TB and STI-related HRVs comprehensively by (1) reducing stigma and discrimination, (2) sensitising and training health and community workers, (3) sensitising lawmakers and law enforcers, (4) launching legal literacy and know your rights campaigns, (5) strengthening legal support services, (6) monitoring, reviewing laws and policies, (7) reducing gender inequality, and (8) addressing gender-based violence.

The South African constitutional, legal and policy framework creates a conducive environment for governmental, non-governmental and private sector stakeholders to ensure the protection and promotion of HIV-related human rights in the country. Additionally, the Prevention and Combating of Hate Crimes and Hate Speech Bill is currently under development. The bill provides grounds for the prosecution of people who commit the offence of hate crime and the offence of hate speech and provides for appropriate sentences that may be imposed on people who commit hate crime and hate speech offences, as well as provides for the prevention of hate crimes and hate speech.^19^

This study has important limitations. First, the uptake of the intervention differed between implementing SR and across districts. Second, there was no routine screening for HRVs among all MSM and TG people seeking HIV prevention services. Therefore, it is likely that the HRVs are under-reported, and the results from this study may not generalise to MSM and TG people in other districts in South Africa. Third, the cross-sectional study design is limited in inferring causal associations.

## Conclusion

Our findings demonstrate the utility and feasibility of screening for and documenting HRVs among MSM and TG people within the context of HIV prevention programmes. The findings suggest the need to systematically screen MSM and TG people of HRVs and link them to legal or other services through a trusted mediator as a standard of care using a rights-based approach that safeguards the dignity and safety of each individual accessing HIV prevention services.

Most of the incidents of HRVs occurred at home, or within family and local community settings, and most were not reported to the authorities for action or mediation. Thus, while the legal basis for redress is necessary, developing the capacity of community-based monitoring systems and structures for mediation is critical for safeguarding and promoting the human rights of MSM and TG people in the country. Finally, addressing HRVs requires adequate funding to support the operationalisation of the comprehensive NSP to reduce human rights-related barriers to HIV and TB services in South Africa 2019–2022 as outlined.
